# High–Performance and Low–Power Complementary Transistors Based on Red Carbon Ribbon

**DOI:** 10.3390/molecules31193365

**Published:** 2026-09-22

**Authors:** Yelu He, Xingyi Tan, Gang Xu, Huan Xiao, Qiang Li

**Affiliations:** 1 Department of Physics, Chongqing Sanxia University of Science and Technology, Wanzhou 404100, China; ghylmy023@163.com (Y.H.); 20040026@sanxiau.edu.cn (G.X.); 20250031@sanxiau.edu.cn (H.X.); 2 College of Intelligent Systems Science and Engineering, Hubei Minzu University, Enshi 445000, China; 2012002@hbmzu.edu.cn

**Keywords:** red carbon ribbon, complementary transistors, sub–5 nm, ab initio method

## Abstract

Due to significant progress in architecture through the vertical stacking of negative channel metal oxide semiconductor (MOS) and positive channel MOS devices, the complementary field–effect transistor (CFET) is regarded as one of the most promising technologies for the next generation beyond the gate–all–around (GAA) architecture. This study comprehensively investigated the transport characteristics of one–dimensional (1D) red carbon ribbon (RCR) field–effect transistors (1D–RCR–FETs) with GAA gate and sub 5 nm gate lengths (*L*_g_), using ab initio methods implemented in the Atomistix ToolKit 2019 software. Based on theoretical simulation results, n– and p–type GAA 1D–RCR–FETs with *L*_g_ of 5 nm satisfied the low–power (LP) and high–performance (HP) criteria for the 2039 horizon, according to the International Roadmap for Devices and Systems (IRDS) 2024 edition, and the corresponding results with *L*_g_ of 3 nm met the HP criteria for the 2039 horizon. Notably, both LP and HP applications demonstrated excellent symmetry between n– and p–FET devices at *L*_g_ = 5 nm, with the power–delay product, delay time, and on–state current ratios often ranging between 0.59 and 2.65, facilitating the development of CFETs. This study provides important theoretical insights highlighting the potential of 1D RCRs as promising channel materials for sub 5 nm 1D CFETs with both HP and LP application requirements.

## 1. Introduction

Currently, the critical dimension of silicon–based field–effect transistors (FETs) has advanced to sub 10 nm and even to sub 5 nm technology nodes [1,2,3,4]. To scale further, implementing innovative device architectures and alternative channel materials is essentially required to lessen the effects of short channels, parasitic resistance, and quantum tunneling [1,2]. From the perspectives of device architecture, the structural optimization of individual devices has approached its physical limitations, progressing from fin–FETs to gate–all–around (GAA) FETs. The complementary FET (CFET) architecture, a novel configuration, is a recent innovation with vertically stacked n– and p–type FETs [5,6], further decreasing the standard cell area and offering new possibilities for vertically integrating logic layers along the *z*–axis [7,8,9,10,11]. However, there is a lack of research into CFETs. In general, one–dimensional (1D) nanoribbons are the channel materials most commonly used for CFETs. Because of their atomic–scale thickness, surfaces free of dangling bonds, and suitability for heterostructure integration, 1D nanoribbon semiconductors have been recognized as viable options to fulfill basic scaling needs [2,3]. Therefore, developing 1D–CFETs, a combination of 1D semiconductors and the advanced CFET architecture, is considered a promising approach to overcome the scaling constraints of silicon devices. To date, efforts to exploit these unique characteristics have focused predominantly on integrating 1D carbon–based materials into FET architectures. Wang et al. [12] pioneered sub 10 nm graphene nanoribbon (GNR) FETs, achieving an on–state current (*I*_on_) density of 2000 μA/μm and an *I*_on_/*I*_off_ (off–state current, *I*_off_) ratio exceeding 10^6^. Senkovskiy et al. [13] subsequently synthesized monolayers comprising multiple aligned heterojunctions of quasi–metallic and wide–bandgap armchair GNRs (AGNRs), exploiting the steep–slope switching of GNR–based tunnel FETs. Martini et al. [14] further investigated FETs employing AGNRs as the channel and graphene sheets as source–drain contacts, observing a clear width–dependent output current. Braun et al. [15] reported 9–AGNR–based FETs with graphene electrodes, attaining an *I*_on_/*I*_off_ ratio of 10^4^ and a maximum *I*_on_ of 70 nA at 1 V. Collectively, these studies establish the viability of 1D carbon–based materials as transistor channels, while underscoring the need to identify systems that combine a suitable bandgap with scalable synthesis. In this context, Odziomek et al. [16] recently synthesized 1D red carbon ribbons (RCRs) exhibiting a bandgap of 1.52 eV, a value well suited to FET operation. Whether RCRs can serve as an effective channel material for CFETs therefore remains a question of both fundamental and practical importance.

This study designed sub 5 nm channel–length GAA FETs using RCRs. Using ab initio quantum transport simulations, the theoretical efficiency of the design in delay time (*τ*), on–state current (*I*_on_), and power–delay product (*PDP*) fulfilled the essential criteria for low–power (LP) and high–performance (HP) applications, in line with the 2039 projections listed in the 2024 International Roadmap for Devices and Systems (IRDS) [17]. The *n*– and *p*–type FETs under conditions of *L*_g_ = 5 nm (underlap, *UL* = 1–3 nm) and *L*_g_ of 3 nm (*UL* = 3 nm) were appropriate for use in HP dissipation devices. Furthermore, the p– and n–type FETs with *L*_g_ of 5 nm (*UL* = 2–3 nm) met the standards of the LP specifications for the IRDS in the 2039 horizon. The p– and n–type FET devices demonstrated a significant, excellent symmetry for *L*_g_ = 5 nm, facilitating the formation of a CFET. The theoretical results demonstrated that 1D RCRs might be a promising channel material candidate for CFETs.

## 2. Results and Discussion

Figure 1a displays the top–view crystal structures of RCRs, yielding the lattice parameter of a = 4.247 Å. According to the PBE functional, the RCRs demonstrated characteristics of a direct bandgap semiconductor with a 1.56 eV bandgap (Figure 1b), which is in agreement with the findings from Odziomek et al. [16], displaying a 1.52 eV bandgap using the same approach, supporting the validity and accuracy of this approach. The energy differences between the Fermi level and the valence band maximum (VBM), and between the Fermi level and the conduction band minimum (CBM), are 0.780 and 0.779 eV, respectively. The *p*_z_ orbitals of the carbon and oxygen atoms dominated CBM and VBM of RCRs, as illustrated in the projected density of states (PDOS, Figure 1b). The PDOS surrounding the VBM and CBM was in line with the findings in the orbital charge distribution, as displayed in the insets of Figure 1b. Based on the equation $\frac{1}{m^{*}}=\frac{1}{\hbar }\frac{d^{2}E}{dk^{2}}$ (m^*^ is effective mass, ℏ is the reduced Planck constant) and fitting the band dispersion from Figure 1b, the electron and hole effective masses for RCRs are calculated as 0.182 and 0.239 *m*_0_, respectively, along the Γ–Z direction, where *m*_0_ is the free–electron mass. Figure 1c,d depict FETs with sub 5 nm *L*_g_ using inherent RCRs as channel materials and strongly n–/p–doped RCRs as electrodes. In theoretical and experimental work, the underlap (*UL*) structure has been shown to improve the device’s performance [18,19,20,21]. The EOT, indicating the thickness of an equivalent oxide layer (SiO_2_, ɛ = 3.9ɛ_0_), was measured to be 0.41 nm, whereas *V*_dd_, the bias voltage, was selected as 0.60 V, according to the IRDS 2024 requirements. The electrodes were doped to introduce additional charges using a compensated background charge [22]. The effect of doping concentration was assessed to analyze the optimal device performance, with the corresponding results presented in Table S1. To reduce subthreshold swing (*SS*) and increase on–state currents, the doping concentrations of N_p_ = 2 × 10^8^ and N_n_ = 4 × 10^8^ m^−1^ were selected for *p*– and *n*–type doping, respectively.

The transfer behaviors of p– and n–type GAA 1D–RCR–FETs with *L*_g_ below 5 nm showed a gradual decrease in all currents with raising voltage (Figure 2). At low voltage, the currents gradually declined with increasing gate voltage (*V*_g_), followed by a sharp decrease with a further increase in *V*_g_, and then slowly decreased with *V*_g_ as the voltage attained a particular value. These specific transport characteristics suggested that these devices typically experience switching effects. To thoroughly illustrate this effect, the *SS* of the GAA 1D–RCR–FETs was determined using the following equation [22,23]: *SS* = ∂*V*_g_/∂(lg*I*_ds_). GAA 1D–RCR–FETs showed considerably decreased *SS* with rising *L_g_* and *UL*, reaching a minimum value of 70 and 79 mV/dec for n– and p–type doping, respectively, as depicted in Figure 3a,b. The obtained values were marginally higher than 60 mV/dec because of the thermionic effect, representing the lower limit observed in traditional FETs. The presence of the *UL* can effectively lower the *SS* by increasing the electrical channel length relative to the metallurgical junction spacing, thereby reducing the influence of the drain potential on the source–side barrier. Quantitatively, the characteristic length (λ) over which the drain field penetrates into the channel scales as $\lambda =\sqrt{\varepsilon_{ch}\cdot t_{ch}\cdot t_{ox}/\varepsilon_{ox}}$, where *ε*_ch_ and *t*_ch_ are the permittivity and thickness of the channel, and *ε*_ox_ and *t*_ox_ are the permittivity and thickness of the gate oxide, respectively. Consequently, the *SS* is suppressed as the effective gate–controlled length increases [18,19,20,21].

The *I*_on_, a key parameter for FETs, corresponds to the current transferred while the *V*_g_ is in the on–state (*V*_g,on_). The bias voltage (*V*_b_) was set at 0.60 V, equivalent to *V*_dd_. The selected off–state current (*I*_off_) values for the HP and LP conditions were 0.01 and 10^−4^ μA/μm, respectively, following the IRDS. Figure 3c,d depict the *I*_on_ values of HP and LP for 1D–RCR–FETs in relation to *L*_g_*,* and the *I*_on_ values and the results are also systematically summarized in Tables S2 and S3. The n–doped 1D–RCR–FETs satisfied the IRDS HP off– and on–state requirements using an *L*_g_ of 5 nm with a *UL* thickness of 1, 2, or 3 nm. Similarly, the above requirement was satisfied at an *L*_g_ of 3 nm and a *UL* thickness of 3 nm. However, only the IRDS HP’s off–state requirements were sufficiently fulfilled for an *L*_g_ of 3 nm with a *UL* thickness of 2 nm. The p–doped 1D–RCR–FETs fulfilled the IRDS HP off– and on–state requirements with various combinations of *L*_g_ and *UL,* showing the suitability of configurations with *L*_g_ of 5 nm (*UL* = 1–3 nm), and *L*_g_ of 3 nm (*UL =* 3 nm). To satisfy the IRDS LP off– and on–state standards, a combination of *L*_g_ = 5 nm with *UL* of 2–3 nm was sufficient for both n– and p–FETs. Furthermore, the off–state requirements satisfied an *L*_g_ of 3 nm with a 3 nm *UL*, meeting the IRDS LP criteria of p–FETs, suggesting that 1D–RCR–FETs are promising for HP and LP applications in n– and p–FETs.

For the GAA RCR–FETs, the effective delay time (*τ*) is described as [24]

*τ* = *C*_t_*V*_dd_/*I*_on_ (1)

where *C*_t_, *C*_g_, and *C*_f_ denote the total, gate, and fringing capacitances, respectively; *C*_t_ = *C*_g_ + *C*_f_; *C*_g_ = ∂*Q*_ch_/∂*V*_g_. Figure 4a,b display the *τ* results with a systematic summary provided in Tables S2 and S3. All the *τ* of GAA 1D–RCR–FETs except n–doped (2 nm *UL* and 3 nm *L*_g_), and *p*–doped (1 nm *L*_g_ and 3 nm *UL*) fulfilled the IRDS requirements to all appearances.

*PDP* is another significant factor contributing to energy change in FET, described by the following equation [25]:

*PDP* = *V*_dd_*I*_on_*τ* (2)

Figure 4c,d present the obtained results, with *PDP* values also systematically summarized in Tables S2 and S3. The *PDP* of GAA 1D–RCR–FETs satisfied the IRDS criteria (0.33 fJ/μm), thereby confirming that 1D–RCR–FETs are suitable for LP and HP devices.

The n– and p–type GAA 1D–RCR–FET implementation with *L*_g_ of 5 nm and *UL* of 3 nm was investigated to gain deeper insights into gate modulation. The local density of states (LDOS) and the spectrum current at different positions during the off– and on–states of the devices is depicted in Figure 5. The electron and hole injection barriers, *Φ*_e_ and *Φ*_h_, are respectively defined as the energy differences between the source/drain chemical potentials (*μ*_S_, *μ*_D_) and the conduction/valence band edges (E_C_, E_V_): *Φ*_e_ = *E*_C_ − *μ*_S_, *Φ*_h_ = *μ*_D_ − *E*_V_. The n–type GAA 1D–RCR–FETs showed an upward band bending with increasing *V*_g_ from −1.0 to −0.3 V, achieving the off–state upon expanding *Φ*_e_ from 0 to 0.31 eV. However, p–type GAA 1D–RCR–FETs demonstrated downward band bending as the *V*_g_ increased from 0.2 to 0.8 V, approaching the off–state when *Φ*_h_ is expanded from 0 to 0.32 eV. These observations are corroborated by the corresponding transmission spectra and transmission eigenstates, as illustrated in Figures S1 and S2. The incident electron wavefunction is effectively evanescent in the off–state, whereas it propagates smoothly into the drain region in the on–states. Notably, it exhibits an opposite phase shift between the p–type and n–type conduction regimes.

The present study aims to analyze the influential factors on I_on_ by examining the interplay between I_on_ and m* based on this work and previous research. Specifically, the investigation focuses on various 1D structures, including the Te/Se/S nanowire [26,27,28], carbon nanotube [29], SbSI nanowire [30], and the Sb_2_Se_3_ nanowire [31], as depicted in Figure 6a. The primary focus of I_on_ is to establish a correlation between the drift velocity of the charge carriers (υ) and the number density of charge carriers (n). The variable υ exhibits an inverse relationship with m*, whereas the density of states (DOS) directly influences n. As depicted in Figure 6a, the variable υ essentially determines the I_on_ for low values of m*. This behavior can be attributed to the fact that a lower effective mass gives rise to greater band curvature and enhanced carrier velocity near the band edge, thereby promoting carrier injection and contributing to a higher I_on_. Then, the I_on_ exhibits an initial decline as the value of m* increases. Subsequently, the significance of the variable n becomes pivotal in I_on_ for elevating m*. In summary, m* and DOS are significant factors that influence the behavior of I_on_s. The electron and hole m* values of the RCR were determined 0.182 and 0.239 m_0_, respectively, as depicted in Figure 1b. The characteristics of I_on_ for the n–type and p–type red carbon FETs exhibit a slight disparity due to their small differing effective masses, as depicted in Figure 6a. The energy efficiency of devices is measured using the energy–delay product (EDP), considering both τ and PDP (EDP = τ × PDP). Figure 6b illustrates the appropriate EDPs for semiconductor FETs with a 1D feature, namely those with a gate length of 5 nm and a UL value of 3 nm; these FETs include the Te/Se/S nanowire [26,27,28], carbon nanotube [29], SbSI nanowire [30], and Sb_2_Se_3_ nanowire [31] semiconductors. The IRDS was used as the prevailing standard for a timeframe up to 2039. Apparently, SbSI and red carbon are the only materials functioning as p–type field–effect transistors (FETs). Specifically, the EDP of p–type red carbon FETs exhibits superior performance to SbSI FETs. The IRDS was effectively fulfilled by utilizing n– and p–doped red carbon ribbon FETs with an L_g_ of 5 nm.

Developing a channel material to deliver symmetric performance in n– and p–type configurations, an essential need for applications of complementary logic, has posed a serious problem for nanomaterial electronics [28,29,30,31]. The results confirmed that optimized gate and supply voltage selection led to remarkable electrical symmetry in 1D–RCR–FETs. Figure 7a–c demonstrate the *I*_on_, τ, and *PDP* symmetry between n– and p–type operations with *L*_g_ of 5 nm and varying *UL*, compared to GaAsH_2_ [32], silicane [33], HfN_2_ [34], InAs [35], HfSnS_3_ [36], BAs [37], MoS_2_ [38], and MoSi_2_N_4_ [39] counterparts. The *I*_on_ (ratio = 0.75) and the *PDP* (ratio = 0.97) displayed optimal symmetry at a *UL* of 1 nm. The τ ratio was always between 1.07 and 1.85, indicating the favorable degree of speed matching for the switch. Furthermore, the HP devices exclusively used HfN_2_ [34], InAs [35], HfSnS_3_ [36] and BAs [37] while the LP application only used monolayer MoS_2_ [38] and MoSi_2_N_4_ [39]. The 1D GaAsH_2_ nanowire [32], 1D RCRs, and the 2D monolayer silicane [33] exhibited suitability for both HP and LP devices. As mentioned above, m* and the DOS are significant factors influencing the behavior of I_on_. First, the electron and hole m* values of the RCR exhibit only a small difference (m_e_* = 0.182 m_0_, m_h_* = 0.239 m_0_), as depicted in Figure 1b. Second, the DOS and the energy differences from the Fermi level to both the VBM and CBM of the RCRs are symmetric, as previously mentioned. In addition, the gate control capability (SS, Figure 3a,b) and the electron and hole injection barriers, *Φ*_e_ and *Φ*_h_ (Figure 5), are also symmetric. These symmetry characteristics confer symmetric behavior on I_on_. Consequently, according to Equations (1) and (2), τ and PDP also exhibit symmetric characteristics. Table S4 summarizes the effective masses and the DOS morphology of low–dimensional GaAsH_2_, silicane, HfN_2_, InAs, HfSnS_3_, BAs, MoS_2_, and MoSi_2_N_4_. Analogous trends were also identified for BAs and InAs, where the effective masses were approximately equal and the DOS exhibited near–perfect symmetry. These symmetry characteristics collectively confirm that sub 5 nm 1D–RCR–FETs are highly promising candidates for advanced CFET technology nodes.

## 3. Model and Approach

All calculations were conducted using the Atomistix ToolKit 2019 software [40]. The drain current was determined using the standard Landauer–Bűttiker relation given below, with details available in Refs. [41,42,43].

(3) $$I\left(V_{b},V_{G}\right)=\frac{2e}{h}\int_{-\infty }^{+\infty }\left\{T\left(E,V_{b},V_{G}\right)\left[f_{S}\left(E-\mu_{S}\right)-f_{D}\left(E-\mu_{D}\right)\right]\right\}\mathrm{dE}$$

Then,

(4) $$T_{k_{//}}\left(E\right)=\mathrm{Tr}[\Gamma_{k_{//}}^{l}(E)G_{k_{//}\;}(E)\;\Gamma_{k_{//}}^{r}\;(E)G_{k_{//}}^{†}(E)\;]$$

where *k*_//_ denotes a surface–parallel approach vector point on a reciprocal lattice at right angles to the transmission direction. The Green function [43,44] below was also used:

(5) $${G_{k}}_{//}(E)=[(E+i\eta^{+})I-{H_{k}}_{//}-\Sigma_{k//}^{S}-\Sigma_{k//}^{D}{]}^{-1}$$

where *H_k//_* and *I* represent the Hamiltonian forms of the channel and identity matrices, respectively, and *η*^+^ and *E* denote modest positive coefficients and energy, respectively. Previous reports indicate that the electron–phonon coupling in light–element materials [45,46,47,48] is weak; accordingly, the phonon scattering effects do not appreciably affect charge transport and are therefore omitted in the present analysis. The Perdew–Burke–Ernzerhof (PBE) functional, which serves as the basis for the Generalized Gradient Approximation (GGA) in describing the exchange–correlation potential [48], i.e., electron exchange and correlation effects in the electronic structure calculations. The atomic positions were completely relaxed until the energy difference for three consecutive steps decreased below 10^−4^ eV, with the residual forces lower than 0.01 eV Å^−1^. Using the Monkhost–Pack k–points approach, the reciprocal space was sampled with an 11 × 1 × 1 grid for geometry optimizations and electronic structure calculations and a 1 × 1 × 100 grid for transport calculations. Doping carrier–induced electron–electron interaction needs to be carefully monitored. Thus, a real–space cutoff energy of 100 Hartree and an electron temperature of 300 K were adopted. The Newman, Newman, and Periodicity boundary conditions are applied in the transverse, vertical, and transport directions, respectively [22].

## 4. Conclusions

Following the recent successful synthesis of red carbon ribbons, the performance capability of sub 5 nm *L*_g_ n– and p–type 1D–RCR–FETs was examined employing the ab initio quantum transport technique for the first time. These FETs revealed favorable *I*_on_, *τ*, and *PDP* characteristics with an appropriate underlap and gate lengths of 5 and 3 nm, meeting the requirement in the IRDS 2024 roadmap, targeting 2039 for LP and HP applications. The results confirmed the key achievement of demonstrating balanced n– and p–type operation, suggesting that RCRs are a promising 1D channel material for CFET implementation. Although interface imperfections and self–heating may degrade device performance in practical nanoscale FETs, our results establish RCRs as a promising 1D channel material for future ultra–scaled, low–power electronics beyond conventional semiconductor technologies.

## Figures and Tables

**Figure 1:**
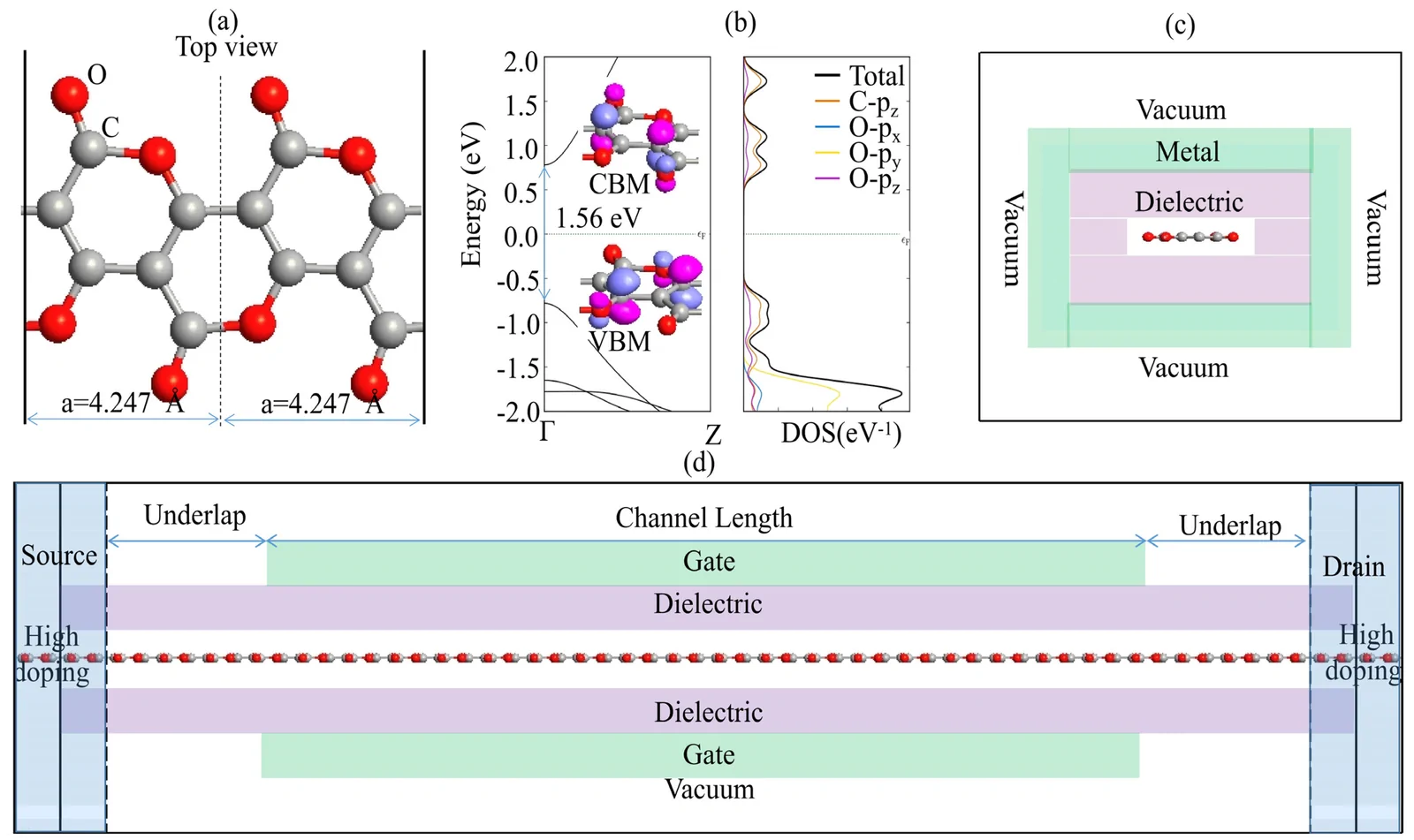
(**a**) Schematic illustrating the structures of the RCRs. (**b**) The band structures of the RCRs. Schematic representing the GAA 1D–RCR–FETs: (**c**) side view and (**d**) cutaway view.

**Figure 2:**
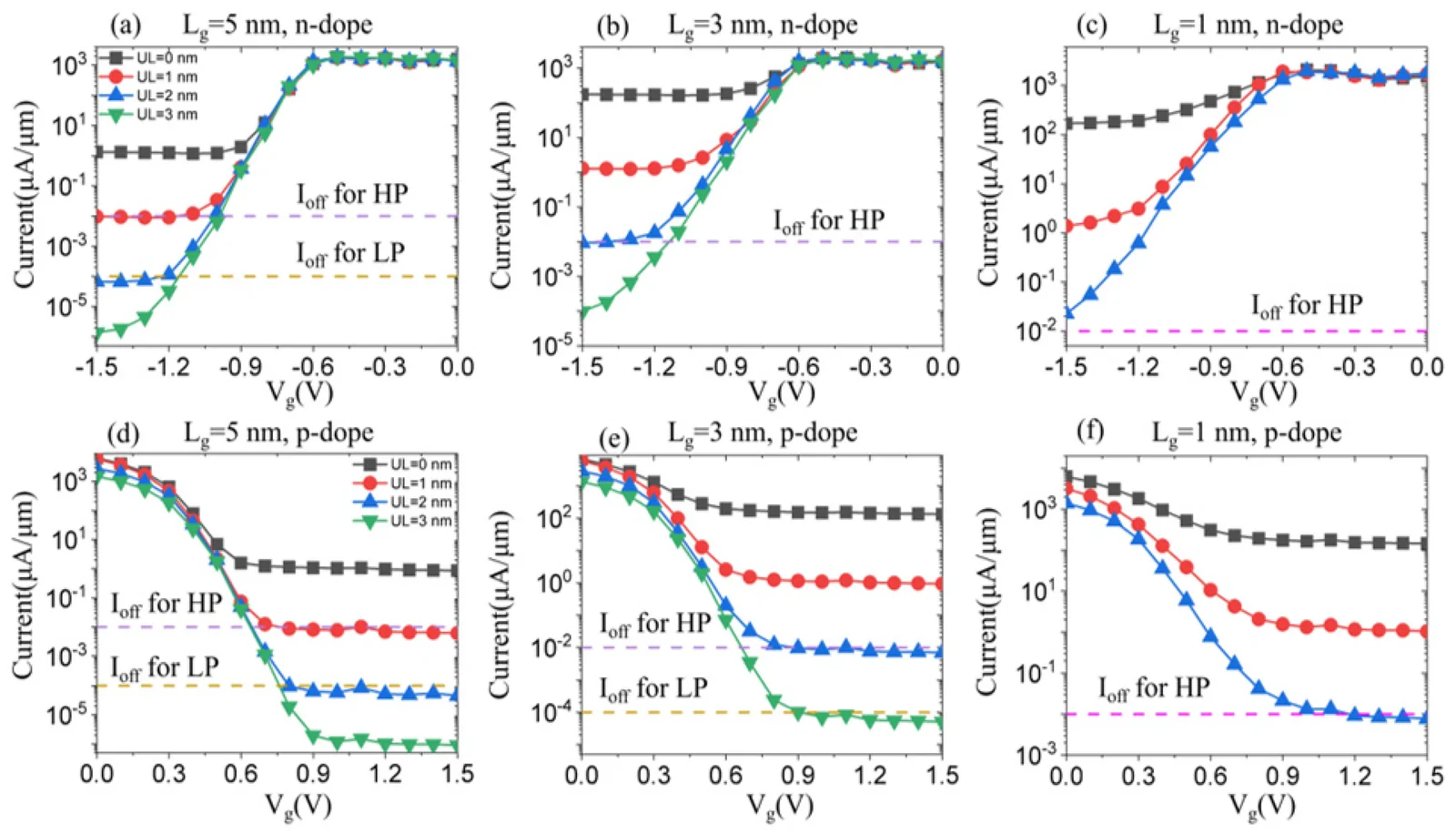
The characteristics associated with the current voltage of GAA 1D–RCR–FETs. Biased voltage: 0.60 V. *UL* values of 0, 1, 2, and 3 nm are indicated by square, circle, upward and downward triangles, respectively.

**Figure 3:**
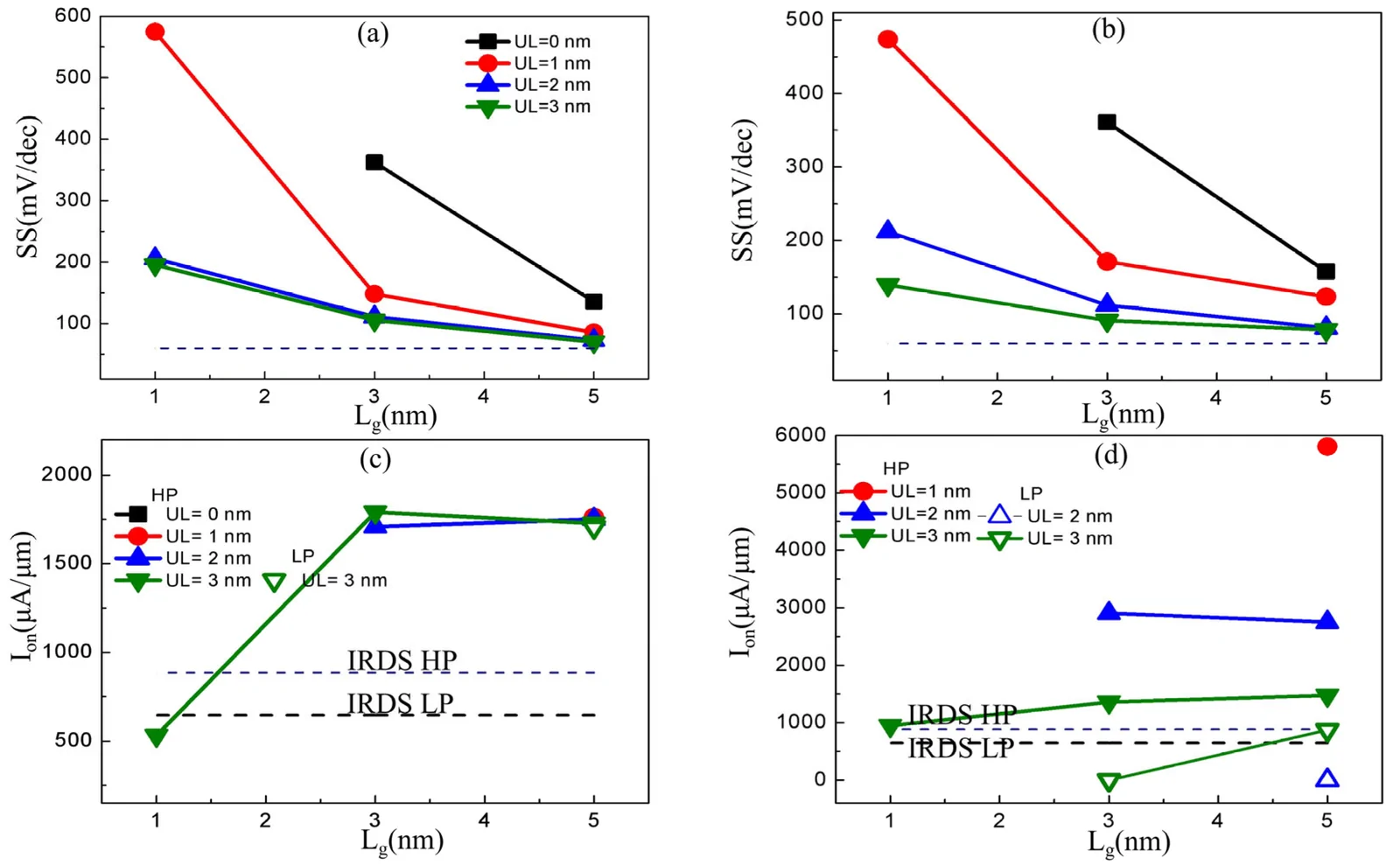
(**a**,**b**) *SS* as a function of *L*_g_ with varying *UL* lengths. (**c**,**d**) *I*_on_ variation of 1D–RCR–FETs with *L*_g_. (**a**,**c**) n–FET; (**b**,**d**) p–FET. The dashed lines denote the benchmark–based adjusted values (*I*_on_) of 885 μA/μm (HP) and 646 μA/μm (LP).

**Figure 4:**
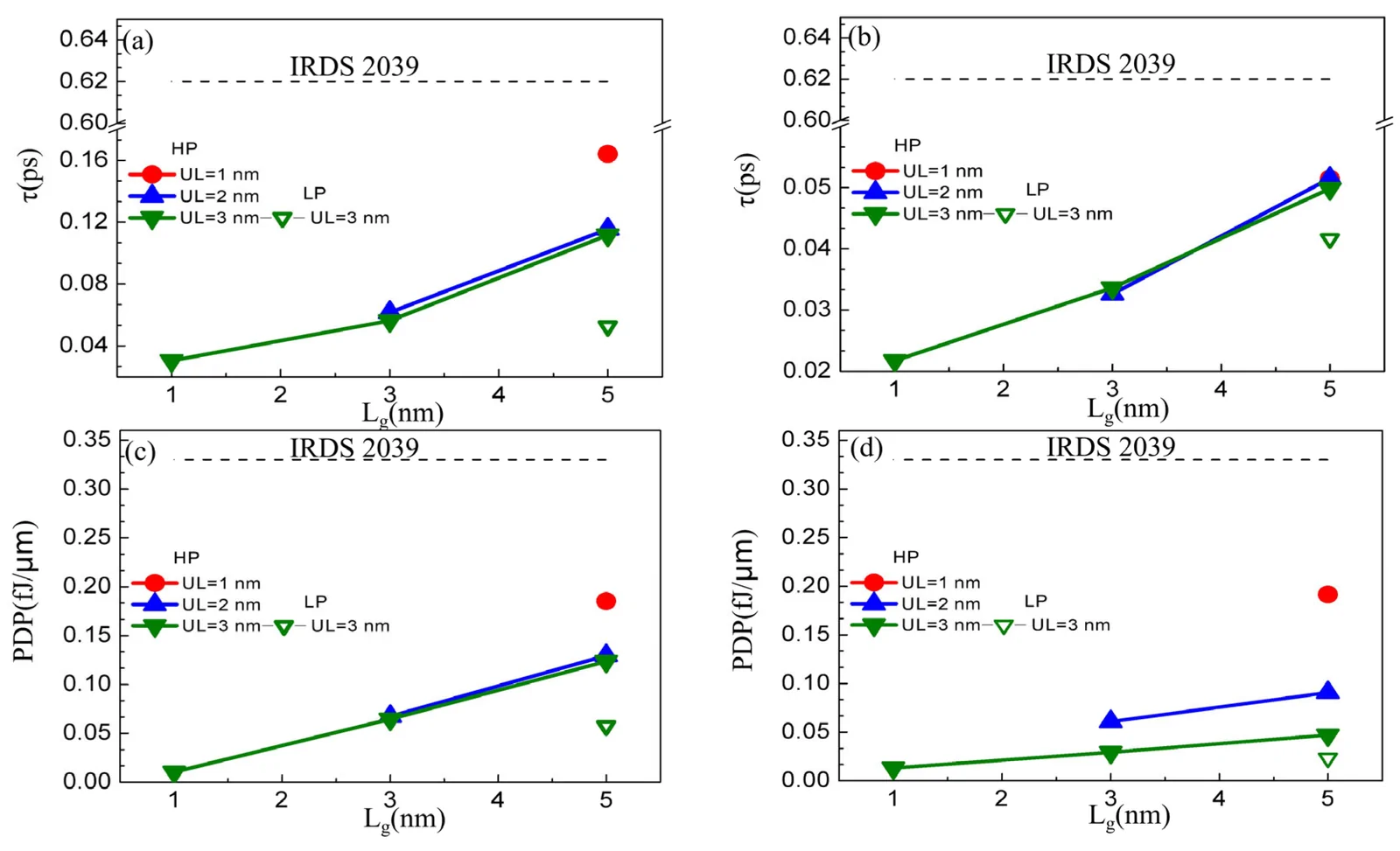
(**a**,**b**) Intrinsic *τ* and (**c**,**d**) PDP versus gate length for 1D–RCR–FETs with varying *L*_g_ values. (**a**,**c**) n–FET; (**b**,**d**) p–FET.

**Figure 5:**
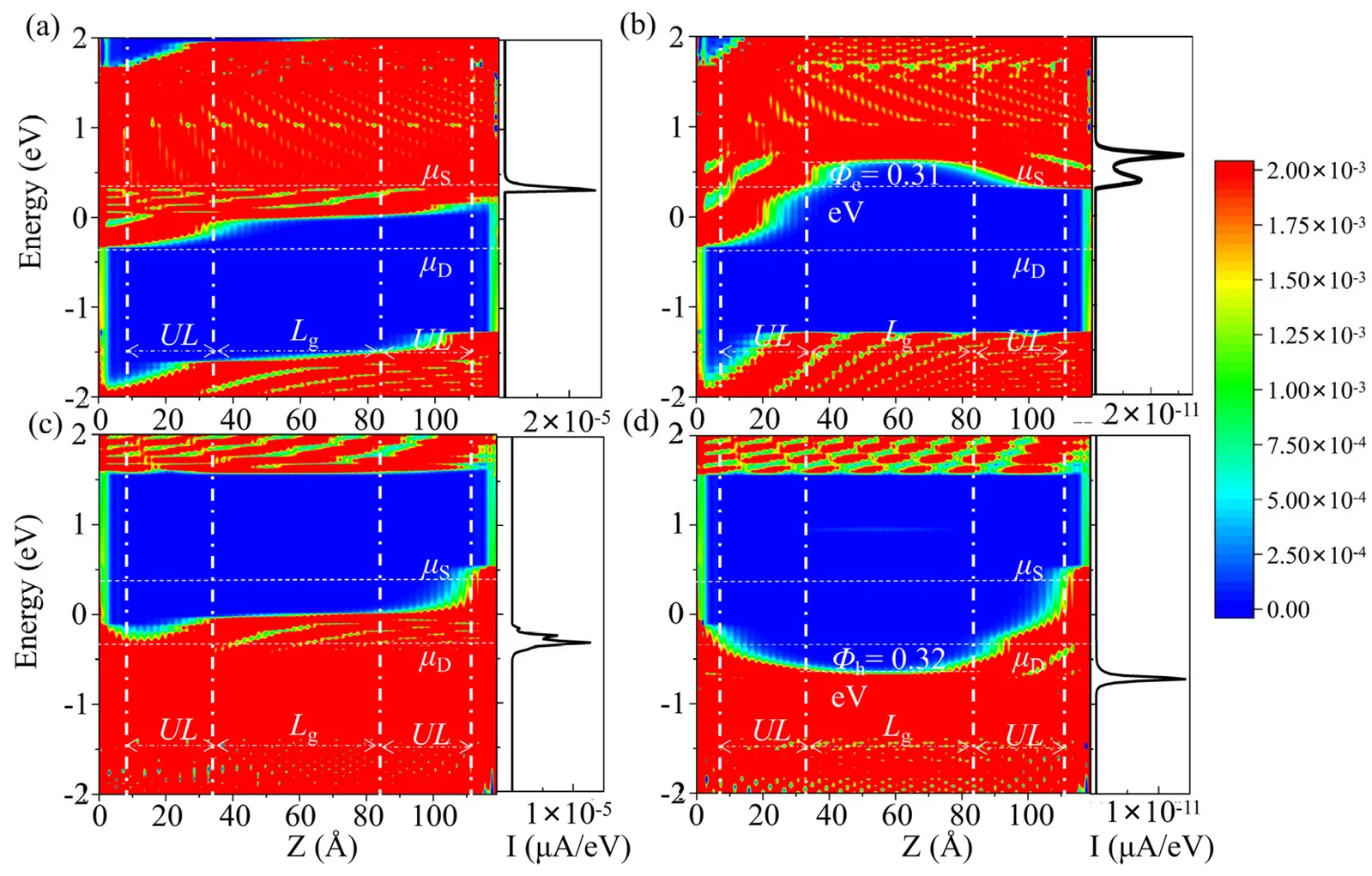
The LDOS and spectral current for *L*_g_ of 5 nm and UL of 3 nm in the on– and off–states. *μ*_s_ and *μ*_d_ denote the electrochemical potentials at the source and drain, respectively. (**a**,**b**) On– and off–state characteristics of *n*–type device; (**c**,**d**) on– and off–state characteristics of p–type device.

**Figure 6:**
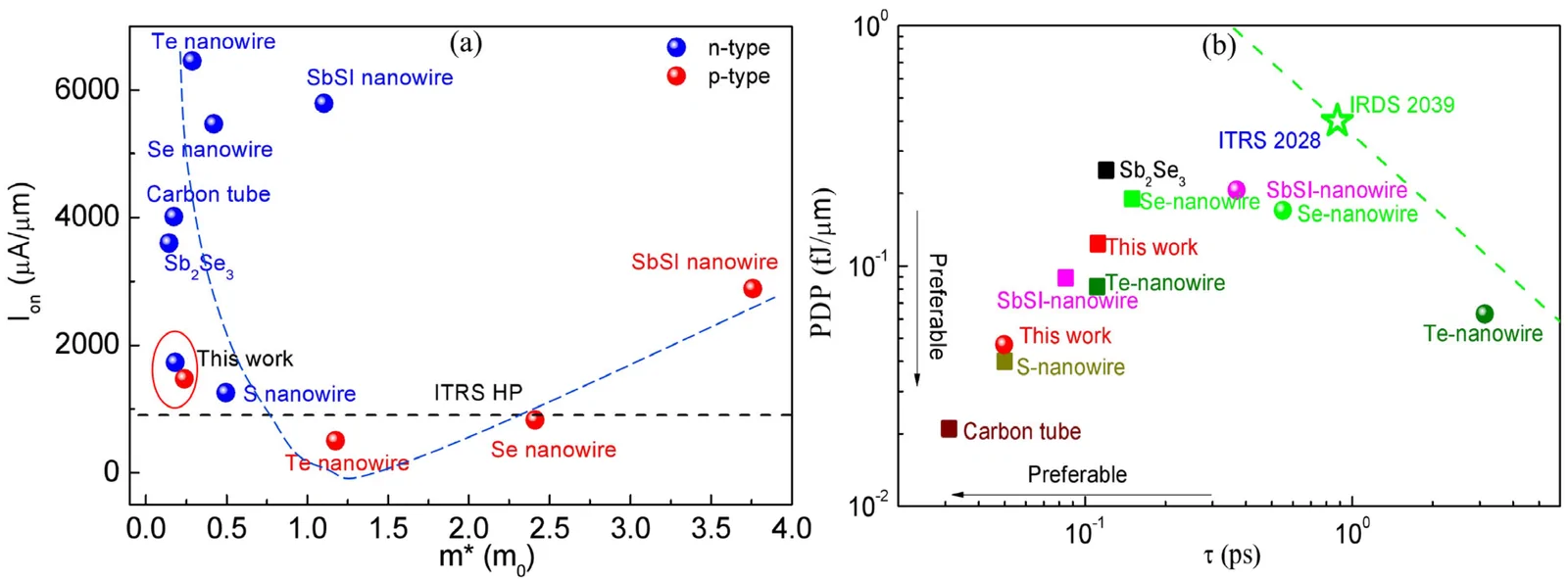
(**a**) The FETs’ on–state current concerning HP usage with L_g_ = 5 nm versus the m* of red carbon ribbon FETs and various one–dimensional channel materials. Ab initio quantum transport simulations were employed to acquire all the data. (**b**) The PDP versus τ of the n–type (square points) and p–type (sphere points) of the 5 nm L_g_ FETs using various channel materials with one–dimensional structures concerning the HP usages. The standard is against the IRDS for the 2039 field of view. PDP = EDP/τ is described by dashed lines.

**Figure 7:**
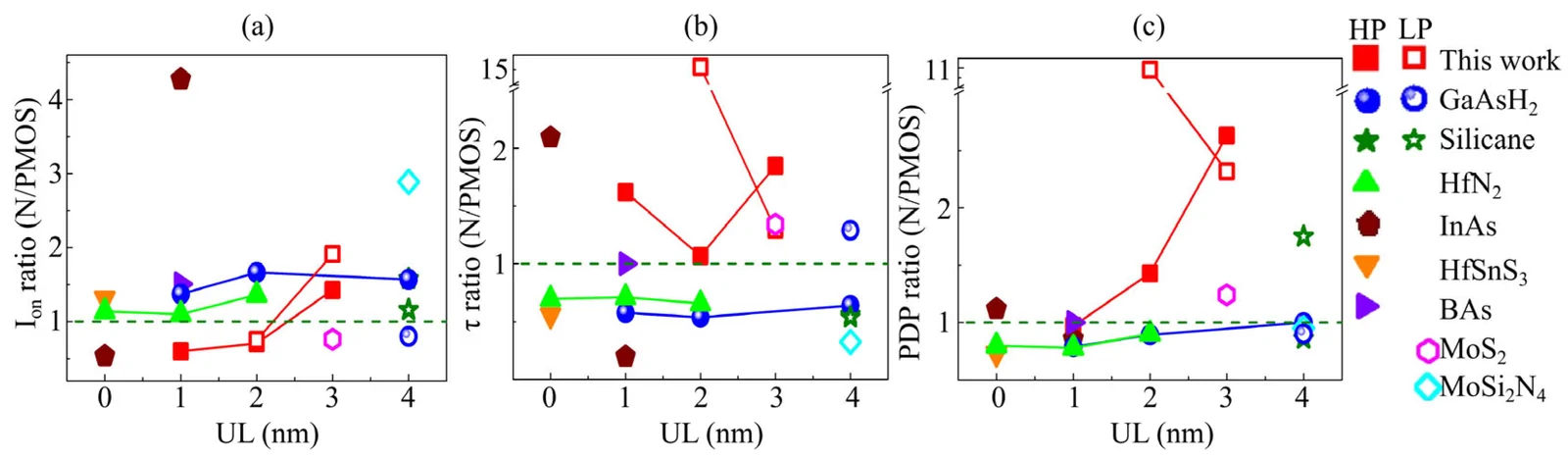
The performance ratios between *n*– and *p*–type FETs. (**a**) I_on_, (**b**) τ, (**c**) PDP.

## Data Availability

Data are openly available in a public repository.

## Supplementary Materials

The following supporting information can be downloaded at https://www.mdpi.com/article/10.3390/molecules31193365/s1. Figure S1. The transmission spectra of the *L*_g_ = 5 nm, *UL* = 3 nm FETs: (a) On-state; (b) off-state of n-type and (c) on-state; (b) off-state of p-type. Figure S2. The transmission eigenstates of the *L*_g_ = 5 nm, *UL* = 3 nm FETs. (a,b) On-state; (c,d) off-state n-type; and (e,f) on-state; (g,h) off-state p-type. Table S1. The doping concentration and the maximum currents. Table S2. Criteria of the sub-5 nm *L*_g_ GAA RCPRs FETs’ ballistic efficiency versus the 2039 requirements of the IRDS 2024 for the HP applications. Table S3. Criteria of the sub-5 nm *L*_g_ GAA RCPRs FETs’ ballistic efficiency versus the 2039 requirements of the IRDS 2024 for the LP applications. Table S4. The effective mass of low dimensional GaAsH_2_, Silicane, HfN_2_, InAs, HfSnS_3_, BAs, MoS_2_ and MoSi_2_N_4_.
