# Supplementary material for: High–Performance and Low–Power Complementary Transistors Based on Red Carbon Ribbon
Source: Molecules. 2026 Sep 22;31(19):3365. doi: 10.3390/molecules31193365 (PMC13666384; doi:10.3390/molecules31193365)
Supplement: Supplementary file 1 [file molecules-31-03365-s001.zip › molecules-4446654-supplementary.pdf]

The Supplemental Material for "High-Performance and Low-Power Complementary Transistors Based on Red Carbon Ribbon"

Yelu He[1], Xingyi Tan[1, 2, §], Gang Xu[1], Huan Xiao[1], Qiang Li[2]

[1]*Department of Physics, Chongqing Sanxia University of Science and Technology, Wanzhou, 404100, China*

[2]*College of Intelligent systems science and engineering, Hubei Minzu University, Enshi, 445000, China*

Contents

1. Figure S1. The transmission spectra of the L_g = *5 nm*, *UL* = 3 *nm* FETs: (a) On-state; (b) off-state of n-type and (c) on-state; (b) off-state of p-type. 2
2. Figure S2. The transmission eigenstates of the L_g = *5 nm*, *UL* = 3 *nm* FETs. (a), (b) On-state; (c), (d) off-state n-type; and (e), (f) on-state; (g), (h) off-state p-type. 2
3. Table S1. The doping concentration and the maximum currents 3
4. Table S2. Criteria of the sub-5 nm L_g GAA RCPRs FETs' ballistic efficiency versus the 2039 requirements of the IRDS 2024 for the HP applications. 4
5. Table S3. Criteria of the sub-5 nm L_g GAA RCPRs FETs' ballistic efficiency versus the 2039 requirements of the IRDS 2024 for the LP applications. 5
6. Table S4. The effective mass of low dimensional $GaAsH_2$, Silicane, HfN_2, InAs, $HfSnS_3$, BAs, MoS_2 and $MoSi_2N_4$. 6

§ Corresponding author E-mail: tanxy@sanxiau.edu.cn

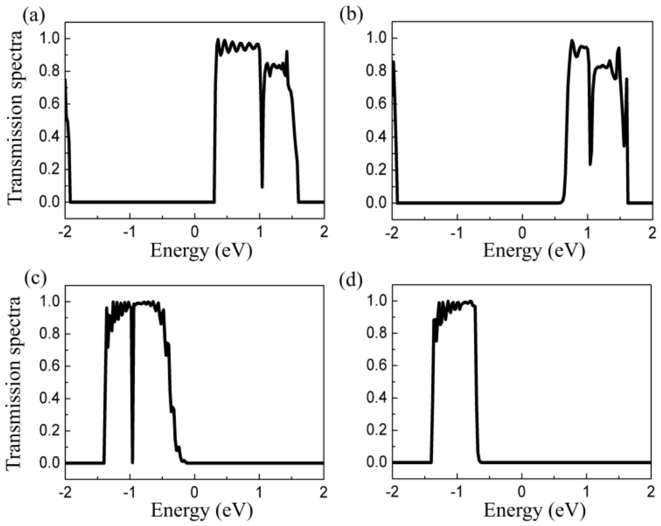

Figure S1. The transmission spectra of the L_g = *5 nm*, *UL* = 3 *nm* FETs: (a) On-state; (b) off-state of n-type and (c) on-state; (b) off-state of p-type.

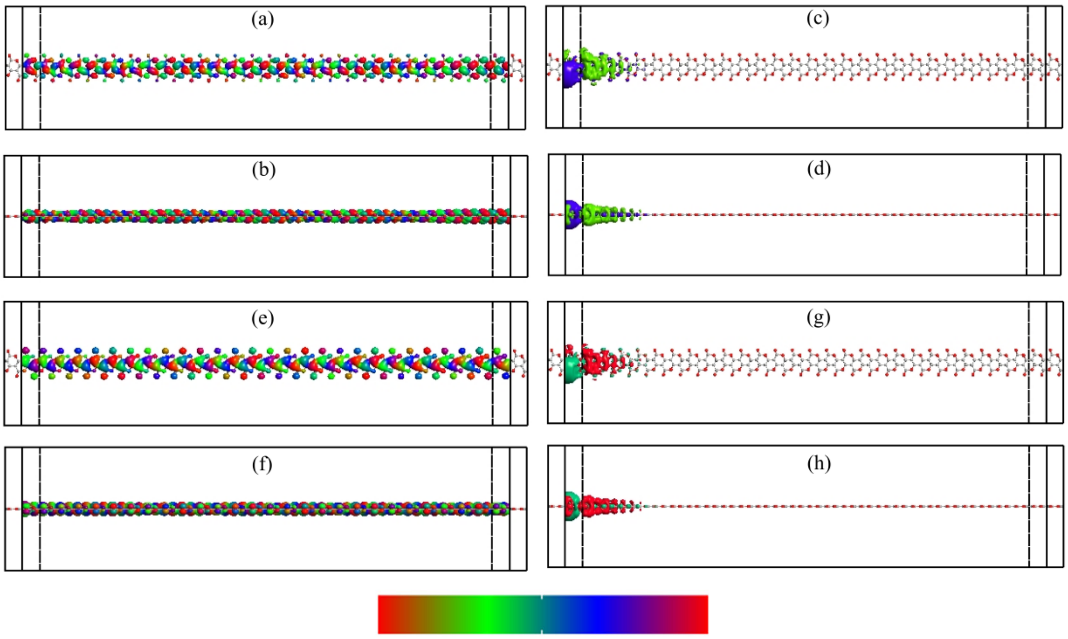

Figure S2. The transmission eigenstates of the L_g = *5 nm*, *UL* = 3 *nm* FETs. (a,b) On-state; (c,d) off-state n-type; and (e,f) on-state; (g,h) off-state p-type.

Table S1. The doping concentration and the maximum currents.

Doping type	Doping concentration (/m)	Maximum currents (μA/μm)
P-type	1×10^8	224.33
	2×10^8	1473.07
	3×10^8	3840.83
N-type	3×10^8	835.74
	4×10^8	1449.90
	5×10^8	2285.47

Table S2. Criteria of the sub-5 nm L_g GAA RCPRs FETs' ballistic efficiency versus the 2039 requirements of the IRDS 2024 for the HP applications.

	L_g (nm)	UL (nm)	*SS* (mV/dec)	I_{off} (μA/μm)	I_{on} (μA/μm)	I_{on}/I_{off}	C_t (fF/μm)	τ (ps)	PDP (fJ/μm)
N-type	5	0	136	0.01	-	-	-	-	-
		1	86	0.01	1318.39	1.32×10^5	0.151	0.206	0.163
		2	73	0.01	1640.42	1.64×10^5	0.105	0.116	0.114
		3	70	0.01	1815.90	1.82×10^5	0.101	0.100	0.109
	3	0	362	0.01	-	-	-	-	-
		1	148	0.01	-	-	-	-	-
		2	111	0.01	62.32	6.23×10^3	0.055	1.585	0.059
		3	105	0.01	1502.26	1.50×10^5	0.053	0.063	0.057
	1	1	574	0.01	-	-	-	-	-
		2	206	0.01	-	-	-	-	-
		3	196	0.01	-	-	-	-	-
P-type	5	0	158	0.01	-	-	-	-	-
		1	124	0.01	2202.03	2.20×10^5	0.156	0.127	0.168
		2	81	0.01	2324.41	2.32×10^5	0.074	0.0109	0.080
		3	79	0.01	1273.02	1.27×10^5	0.038	0.054	0.041
	3	0	361	0.01	-	-	-	-	-
		1	171	0.01	-	-	-	-	-
		2	112	0.01	371.38	3.71×10^4	0.049	0.240	0.053
		3	91	0.01	1037.23	1.04×10^5	0.024	0.041	0.026
	1	1	474	0.01	-	-	-	-	-
		2	212	0.01	-	-	-	-	-
		3	140	0.01	1.18	1.18×10^2	0.011	16.379	0.012
IRDS HP 2039	10	-	70	0.01	885	8.85×10^4	0.31	0.62	0.33

L_g: the gate length. *UL*: the underlap length. *SS*: the subthreshold swing. I_{off}: the off-state current. I_{on}: the on-state current. C_g: the gate capacitance. τ: the delay time. *PDP*: the power dissipation.

Table S3. Criteria of the sub-5 nm L_g GAA RCPRs FETs' ballistic efficiency versus the 2039 requirements of the IRDS 2024 for the LP applications.

	**L_g (nm)**	**UL (nm)**	**SS (mV/dec)**	**I_{off} (μA/μm)**	**I_{on} (μA/μm)**	**I_{on}/I_{off}**	**C_t (fF/μm)**	**τ (ps)**	**PDP (fJ/μm)**
**N-type**	5	0	136	10^{-4}	-	-	-	-	-
		1	86	10^{-4}	-	-	-	-	-
		2	73	10^{-4}	728.04	7.28×10^{6}	0.128	0.317	0.138
		3	70	10^{-4}	1342.80	1.34×10^{7}	00.47	0.063	0.051
	3	0	362	10^{-4}	-	-	-	-	-
		1	148	10^{-4}	-	-	-	-	-
		2	111	10^{-4}	-	-	-	-	-
		3	105	10^{-4}	-	-	-	-	-
	1	1	574	10^{-4}	-	-	-	-	-
		2	206	10^{-4}	-	-	-	-	-
		3	196	10^{-4}	-	-	-	-	-
**P-type**	5	0	158	10^{-4}	-	-	-	-	-
		1	124	10^{-4}	-	-	-	-	-
		2	81	10^{-4}	968.99	9.69×10^{6}	0.012	0.022	0.013
		3	79	10^{-4}	703.03	7.03×10^{6}	0.019	0.048	0.022
	3	0	361	10^{-4}	-	-	-	-	-
		1	171	10^{-4}	-	-	-	-	-
		2	112	10^{-4}	-	-	-	-	-
		3	91	10^{-4}	152.17	1.52×10^{6}	0.009	0.103	0.009
	1	1	474	10^{-4}	-	-	-	-	-
		2	212	10^{-4}	-	-	-	-	-
		3	140	10^{-4}	-	-	-	-	-
IRDS LP 2039	10	-	65	10^{-4}	646	6.46×10^{6}	0.31	0.62	0.33

L_g: the gate length. *UL*: the underlap length. *SS*: the subthreshold swing. I_{off}: the off-state current. I_{on}: the on-state current. C_g: the gate capacitance. τ: the delay time. *PDP*: the power dissipation.

Table S4. The effective mass of low dimensional $GaAsH_2$, Silicane, HfN_2, InAs, $HfSnS_3$, BAs, MoS_2 and $MoSi_2N_4$.

	Electron effective mass (m_0)	Hole effective mass (m_0)	DOS symmetrical	References
$GaAsH_2$	0.09	0.53	No	Ref. 32
Silicane	0.118	3.209	No	Ref. 33
HfN_2	0.575	0.766	No	Ref. 34
InAs	0.17	0.23	approximate	Ref. 35
$HfSnS_3$	0.24	4.64	No	Ref. 36
BAs	0.47	0.43	approximate	Ref. 37
MoS_2	0.43	0.53	No	Ref. 38
$MoSi_2N_4$	0.428	0.487	No	Ref. 39

32. Li, Q.; Fang, S.; Liu, S.; Xu, L.; Xu, L.; Yang, C.; Yang, J.; Shi, B.; Ma, J.; Yang, J.; et al. Performance limit of ultrathin GaAs transistors. *ACS Appl. Mater. Interfaces* **2022**, *14*, 23597–23609.

33. Pan, Y.; Dai, J.; Xu, L.; Yang, J.; Zhang, X.; Yan, J.; Li, J.; Shi, B.; Liu, S.; Hu, H.; et al. Sub−5−nm monolayer silicane transistor: A first−principles quantum transport simulation. *Phys. Rev. Appl.* **2020**, *14*, 024016.

34. Liu, X.; Mao, Y. Single−layer HfN_2: Symmetric scaling behavior in CMOS transistors. *J. Mater. Chem. C* **2025**, *13*, 16628.

35. Li, Q.; Yang, C.; Xu, L.; Liu, S.; Fang, S.; Xu, L.; Yang, J.; Ma, J.; Li, Y.; Wu, B. Symmetric and Excellent Scaling Behavior in Ultrathin n−and p−Type Gate−All−Around InAs Nanowire Transistors. *Adv. Funct. Mater.* **2023**, *33*, 2214653.

36. Chen, C.; Yang, J.; Qu, H.; Zhou, W.; Guo, T.; Sun, W.; Song, X.; Yuan, X.; Zhang, S. Symmetric high−performance transport in n−and p−type $HfSnS_3$ nanowire gate−all−around transistors toward sub −5 nm electronics. *Phys. Rev. Appl.* **2025**, *23*, 044002.

37. Li, Q.; Zheng, T.; Xu, L.; Fang, S.; Yang, Z.; Xu, L.; Li, Y.; Wu, B.; Yang, X.; Quhe, R.; et al. N−and P−type symmetric scaling behavior of monolayer hydrogenated boron arsenide transistors. *Phys. Rev. Mater.* **2024**, *8*, 014603.

38. Zhang, H.; Shi, B.; Xu, L.; Yan, J.; Zhao, W.; Zhang, Z.; Lu, J. Sub−5 nm monolayer MoS_2 transistors toward low−power devices. *ACS Appl. Mater. Interfaces* **2021**, *3*, 1560–1571.

39. Huang, J.; Li, P.; Ren, X.; Guo, Z. Promising properties of a sub−5−nm monolayer $MoSi_2N_4$ transistor. *Phys. Rev. Appl.* **2021**, *16*, 044022.
